# Characterization of Human γδ T Lymphocytes Infiltrating Primary Malignant Melanomas

**DOI:** 10.1371/journal.pone.0049878

**Published:** 2012-11-26

**Authors:** Adriana Cordova, Francesca Toia, Carmela La Mendola, Valentina Orlando, Serena Meraviglia, Gaetana Rinaldi, Matilde Todaro, Giuseppe Cicero, Leonardo Zichichi, Paolo Li Donni, Nadia Caccamo, Giorgio Stassi, Francesco Dieli, Francesco Moschella

**Affiliations:** 1 Dipartimento di Discipline Chirurgiche ed Oncologiche, Università di Palermo, Palermo, Italy; 2 Dipartimento di Biopatologia e Biotecnologie Mediche e Forensi, Università di Palermo, Palermo, Italy; 3 Unità Operativa di Dermatologia, Azienda Ospedaliera “S. Antonio Abate”, Trapani, Italy, 4 Dipartimento di Scienze Economiche, Aziendali e Finanziarie, Università di Palermo, Palermo, Italy; University of Tennessee, United States of America

## Abstract

T lymphocytes are often induced naturally in melanoma patients and infiltrate tumors. Given that γδ T cells mediate antigen-specific killing of tumor cells, we studied the representation and the *in vitro* cytokine production and cytotoxic activity of tumor infiltrating γδ T cells from 74 patients with primary melanoma. We found that γδ T cells represent the major lymphocyte population infiltrating melanoma, and both Vδ1^+^ and Vδ2^+^ cells are involved. The majority of melanoma-infiltrating γδ cells showed effector memory and terminally-differentiated phenotypes and, accordingly, polyclonal γδ T cell lines obtained from tumor-infiltrating immune cells produced IFN-γ and TNF-α and were capable of killing melanoma cell lines *in vitro*. The cytotoxic capability of Vδ2 cell lines was further improved by pre-treatment of tumor target cells with zoledronate. Moreover, higher rate of γδ T cells isolation and percentages of Vδ2 cells correlate with early stage of development of melanoma and absence of metastasis. Altogether, our results suggest that a natural immune response mediated by γδ T lymphocytes may contribute to the immunosurveillance of melanoma.

## Introduction

Melanoma is one of the more frequently studied cancers for the evaluation of immunotherapy, and with its epidemic rise in incidence across the world, it has become the focus of multiple immunotherapy trials over the years. The evidences that spontaneous regression of disease may occur in patients with melanoma [1]–[3] and that increased numbers of tumor-infiltrating lymphocytes (TILs) correlate to improved prognosis of melanoma and other solid tumors [4], [5] have argued for the importance of the immune response in the clinical outcome of melanoma. In addition, metastatic melanoma has a poor prognosis with a five-year survival of less than 2% and very limited treatment options. Approved treatments for metastatic melanoma include high-dose interleukin-2 (IL-2) and chemotherapy [6], [7], which have, at best, an overall response rate of 16% and 7.5%, with very few complete responders and almost no long-term survivors [7].

TILs are an immune population composed of different immune cells that have specificity and potential reactivity against the tumor. TILs have been found in a wide variety of solid tumors including primary brain tumors, epithelial cancers, melanoma and others [8]–[10]. Recent studies have reported high clinical response rates in metastatic melanoma patients with TIL-based protocols in combination with non-myeloablative lymphodepleting chemotherapy (cyclophosphamide and fludarabine) immediately prior or added to infusion of TIL and high-dose IL-2 therapy [11]–[13]. Other novel strategies include peptide vaccination and blocking antibodies against the CTLA-4 [14]–[16] or the PD-1 molecule [17]–[19]. Clearly, combination therapy with other agents or perhaps modification of TILs has the potential to further improve clinical responses, decrease the number of TILs needed for therapy and/or decrease the toxicities associated with cyclophosphamide/fludarabine preparative regimens.

While the functions and anti-melanoma activities of CD4 and CD8 T cells within TILs have been extensively studied [20]–[21], very little is known about γδ T lymphocytes. γδ T lymphocytes are important effector cells of the immune system that may play a role in the anti-tumor surveillance in the periphery [22]. Human γδ T cells can be divided into two main populations based upon δ chain expression [23]: γδ T cells expressing the Vδ1 chain are most often found in mucosal tissues, where they are involved in maintaining epithelial tissue integrity in the face of damage, infection, or tumor transformation, while γδ T cells expressing the Vδ2 chain predominate in the peripheral blood and secondary lymphoid organs [24]. While the ligand(s) recognized by Vδ1^+^ cells remain unknown, Vδ2^+^ cells recognize non peptidic antigens by a MHC-unrestricted mechanism, an important feature which distinguishes them from αβ T cells [23]. Specifically, Vδ2 T cells recognize phosphoantigens that are produced through the isoprenoid biosynthesis pathways [25]–[27]. Phosphoantigens are not stimulatory at physiologic levels, but transformed and infected cells, produce increased levels of metabolic intermediates that are able to activate Vδ2 T cells [28]–[30]. Accordingly, Vδ2 T cells can also be activated, through an indirect mechanism, by aminobisphosphonates, a class of drugs used to treat certain bone diseases, that inhibit farnesyl pyrophosphate synthase, and cause accumulation of endogenous upstream metabolites such as isopentenylpyrophosphate (IPP) [31].

γδ T cells may indirectly contribute to the immune defense against cancer cells, by producing cytokines typical of Th1, Th2 or Th17 cells [32]–[34], or cross-talking with dendritic cells [35], macrophages [36] and B cells [37]–[38]. Additionally, γδ T cells perform cytotoxic activity toward cancer cells, which is mediated in much the same manner as for αβ T cells, through perforin/granzyme, Fas/FasL, TNF/TNF-R and TRAIL-TRAIL-R pathways [24]. Moreover, the localization of γδ T cells within epithelia suggests that these cells may contribute to the surveillance of malignancies, including melanoma [22]. Based on this, we evaluated the representation and the *in vitro* cytokine production and cytotoxic activity of tumor infiltrating γδ T cells from 74 patients with primary melanoma. Moreover, we correlated levels of infiltrating γδ T cells and any of the established clinicopathologic features described for melanoma.

## Materials and Methods

### Patients

We identified a consecutive series of patients who were treated surgically at the Plastic Surgery Department of the University Hospital in Palermo, from 2007 to 2010, for primary melanoma and for whom fresh-frozen tissue and peripheral blood were available for evaluation. A total of 74 patients (32 females and 42 males) were included and all were followed postoperatively for the development of important clinical outcomes such as metastasis and death. The median age at surgery was 60 years (range 26–80 years). Healthy subjects were volunteers in good and stable clinical condition, and had laboratory parameters in the physiologic range. Diagnosis of melanoma was histologically confirmed.

Melanoma morphology was superficial spreading (46%), nodular (35%), acral lentiginous (5%), lentigo maligna (3%), spitzoid (3%), and nevoid (1%).

All patients brought cutaneous primary melanoma and were staged according to the new American Joint Committee on Cancer staging system for cutaneous melanoma [39]. A blood drawing was taken before the surgical excision. According to Italian rules (art. 13, DLgs n. 196/03), this study did not require authorisation by the local ethical committee. The study was performed in accordance to the principles of the Helsinki declaration and all individuals gave written informed consent to participate.

### Isolation of Tumor-infiltrating Immune Cells and PBMCs and FACS Analysis

Peripheral blood mononuclear cells (PBMC) were obtained by density gradient centrifugation using Ficoll-Hypaque (Pharmacia Biotech, Uppsala, Sweden). Tissue specimens were obtained from 74 different patients undergoing standard-of-care surgical procedures for cutaneous melanoma, at the time of primary surgery. There were no restrictions (e.g. stage, etc) on tissues included for this study other than confirmation of melanoma by pathology review of H&E slides from the specimen taken for research. Tissue was obtained fresh and immediately transported to the laboratory in sterile saline for processing. Tissue was first minced into small pieces followed by digestion with collagenase type IV and DNAase (Sigma, St. Louis, MO) for 1 hr at 37°C. After digestion, the cells extracted were washed twice in RPMI 1640 medium (Gibco, Grand Island, NY, USA) and tumor-infiltrating mononuclear cells were obtained by Ficoll-Hypaque density gradient centrifugation. Both PBMC and tumor-infiltrating cells were stained for live/dead discrimination using Invitrogen LIVE/DEAD fixable violet dead cell stain kit (Invitrogen, Carlsbad, CA). Fc receptor blocking was performed with human immunoglobulin (Sigma, 3 µg/ml final concentration) followed by surface staining with different fluorochrome-conjugated antibodies to study the composition of the different subpopulations.

The fluorescein isothiocyanate (FITC)-, phycoerythrin (PE)-, PE-Cy5- or allophycocyanin (APC)- conjugated monoclonal antibodies (mAbs) used to characterized the entire population were the following: anti-CD3, anti-CD4, anti-CD8, anti-CD14, anti-CD19, anti-CD56, anti-pan γδ TCR, anti-Vδ1, anti-Vδ2, anti-CD27 and anti-CD45RA, all purchased from BD Biosciences (Mountain View, CA). Expression of surface markers was determined by flow cytometry on an FACSCalibur or FACSCanto II Flow Cytometer with the use of FlowJo software (BD Biosciences). The gating strategy involved progressively measuring total cells; viable cells only; lymphomonocytes and specific cell types. For every sample 100.000 nucleated cells were acquired and values are expressed as percentage of viable lymphomonocytes, as gated by forward and side scatter. Negative control (background) values were not subtracted, as the median backgrounds for isotype-matched mAbs was 0.0028% (range, 0.000%–0.0063%). Samples were considered positive if the number of cells was equal to or greater than 0.01% and at least 10 clustered events were apparent. This empiric cut-off value was predicted to be >90% different from background, at an α of 0.05 [40].

### Generation of Polyclonal Vδ1^+^ and Vδ2^+^ T Cell Lines

Polyclonal Vδ1^+^ and Vδ2^+^ T cell lines were generated by first enriching bulk tumor infiltrating cells using a γδ T cell isolation kit (Miltenyi Biotec, Bergisch Gladbach, Germany), followed by sorting single Vδ1^+^ and Vδ2^+^ γδ T cell through a FACSAria (BD Biosciences) with specific mAbs. Cells (2×10^3^) were then cultured into each well of round-bottom, 96-well plates containing 2×10^4^ irradiated (40 Gy) allogeneic PBMC, 2×10^3^ irradiated (70 Gy) EBV-transformed B cells, 0.5 µg/ml PHA, and 200 U/ml recombinant interleukin 2 (Proleukin; Novartis Pharma, Basel, Switzerland). Growing lines were expanded in 200 U/ml IL-2 and restimulated every 2 weeks. Usually, cells were collected after 4–6 weeks of culture to be used for functional assays *in vitro*.

### Cytokine Production and Cytotoxic Assay

The cytokine production capacity of polyclonal Vδ1^+^ and Vδ2^+^ T cell lines was determined after stimulation with PMA (BD Biosciences, 20 ng/ml) and ionomycin (BD Biosciences, 1 µM) for 24 h at a cell concentration of 10^6^/ml. ELISA kits for the detection of IFN-γ and TNF-α were obtained from BD Pharmingen and performed according to the manufacturer’s instructions.

The human malignant melanoma A375 cell lines (a generous gift of Dr. Ruggero De Maria, Istituto Superiore di Sanità, Rome, Italy), growing in adhesion in Dulbecco’s Modified Eagle’s Medium (DME) with 10% FCS, was used as target in cocultures with polyclonal Vδ1^+^ and Vδ2^+^ T cell lines obtained from 8 patients affected by melanoma. Briefly, Vδ1^+^ and Vδ2^+^ T cells resuspended at the final concentrations of 10^6^ and 2.5×10^6^ cells/ml were added to A375 cells (1×10^5^), to obtain the E:T ratios of 10∶1 and 25∶1. Cocultures were maintained for 6 hrs a 37°C in presence of 5% of CO_2_. In some experiments, tumor cells were pre-treated overnight with Zoledronate (Novartis Pharma, Basel, Switzerland, 5 µM final concentration), before incubation with Vδ1^+^ or Vδ2^+^ T cell lines. At the end of incubation, the cells were washed with PBS and stained with 10 µl of Annexin V FITC and 10 µl of PI solution in 100 µl of 1× binding buffer (10 mM HEPES, 140 mM NaCl, and 25 µM CaCl_2_) for 15 min at room temperature in the dark, diluted in 400 µl 1× binding buffer, and immediately analysed by FACS (10,000 events/sample) to establish the level of cells death. Samples were acquired on an FACSCalibur and the cells were gated by forward and side scatter on A375 melanoma target cells. The evaluation of cytotoxic activity was based on the degree of reduction of viable target cells (VTC) analyzed through the expression of Annexin V and propidium iodide (Annexin V^−^ PI^−^).

### Immunohistochemistry

Immunohistochemical analysis was performed on 5 µm-thick paraffin-embedded sections of melanoma samples. Slides were heated for antigen retrieval in 10 mM sodium citrate (pH 6.0). After rinsing in dH_2_O, inhibition of endogenous peroxidase was performed with 3% H_2_O_2_ (5 min). After two washes in TBS, slides were incubated with 10% human serum for 20 min to clock unspecific staining. Following elimination of excess serum, sections were then exposed to specific antibodies against anti-human γδ TCR (purified mouse anti-human γδ TCR, IgG1, BD Pharmingen) or isotype-matched controls at appropriate diluitions, overnight at 4°C. Then sections were washed in TBS and incubated with biotinylated anti-mouse antibody for 30 min at room temperature and treated with streptavidin-peroxidase (LSAB2 Kit, Dako). Staining was revealed using AEC substrate and counter-stained with hematoxylin. Positive controls for γδ TCR staining consisted of normal human lymph node tissue resected for diagnostic purposes.

### Statistical Analysis

The significance of difference between groups was determined by Student’s t test. Pearson χ^2^, likelihood-ratio χ^2^, Fischer’s exact test statistics and a simple logistic regression analysis were applied to compare the effect of cancer stage on the recovery rate and percentage of γδ T cells. Differences between subgroups with a probability of <0.05 were regarded as significant. All p-values are two tailed and all statistical analyses were performed using the STATA software package.

## Results

### Phenotype of Melanoma Infiltrating Immune Cells

**Figure 1:**
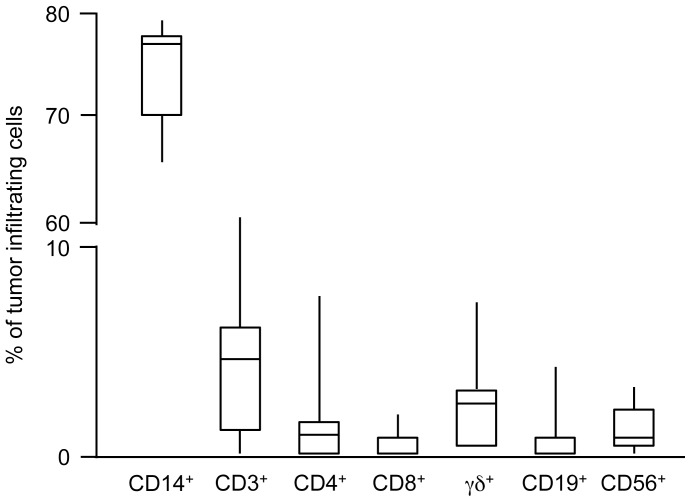
Percentages of tumor-infiltrating immune cells in patients with primary melanoma. Box plots of percentages of immune cell subsets in 74 melanoma patients. Boxes represent 25^th^ to 75^th^ percentiles. Middle bar identifies median; whiskers show minimum and maximum.

After surgical removal of 74 primary cutaneous melanomas, tumor-infiltrating immune cells were isolated only in 54 of them (72.9%), and their subpopulations were studied. The greatest numbers of infiltrating immune cells were macrophages (CD14^+^ cells), while CD3^+^ T cells were scarcely represented within the analyzed primary melanomas (4.3%). γδ T cells were the major lymphocyte subset and accounted for approximately 50% of the total CD3^+^ population (**Figure 1**). Of note however, γδ T cells were found only in 46 out of the 54 patients with a lymphocyte infiltration. Frequencies of CD4^+^ and CD8^+^ T cells in tumor-infiltrating immune cells were 37% and 13% of the CD3^+^ population, respectively, and percentages of B and NK lymphocytes were even lower than percentages of CD3^+^ cells (1.2% for both CD19^+^ and CD56^+^ cells). As shown in **Figure 1**, there was an extremely high variability in percentages of cells recovered from the tested melanoma patients.

### Distribution and Phenotypic Analysis of Melanoma-infiltrating γδ T Cells

**Figure 2:**
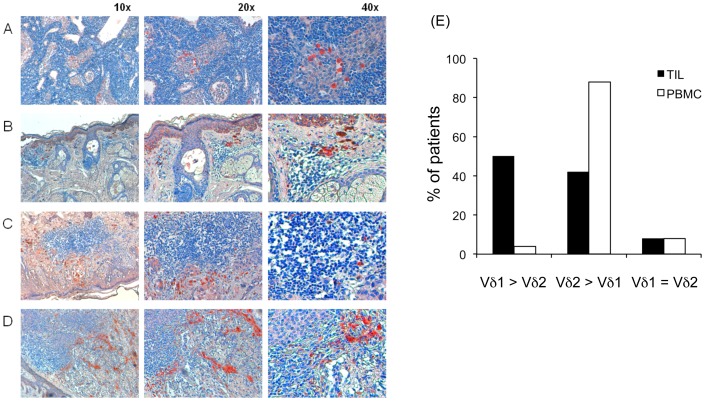
Localization and subset phenotype of melanoma-infiltrating γδ T cells. (A–D) immunohistochemistry staining of melanoma tissues using antibodies directed against the γδ TCR, as revealed by AEC (red staining). Nuclei are revealed by hematoxylin staining (blue). (A) shows lymph node staining for γδ TCR, as a positive control. Different histotypes of melanoma are represented: superficial intraepithelial melanoma (B), superficial spreading melanoma (C) and ulcerated nodular melanoma infiltrating the dermis (D). Melanomas with moderate amounts of γδ^+^ tumor-infiltrating lymphocytes (B and C) and high (D) levels of γδ^+^ T cells are shown. In all cases, γδ^+^ T cells are distributed in the periphery of the infiltrate. (E) Percentages of total γδ T cells, and their Vδ1 and Vδ2 subsets in TILs and PBMCs of patients with melanoma.

To evaluate the distribution of γδ T cells within melanomas, we immunostained melanoma sections from 7 patients for pan-γδ TCR. As shown in **Figure 2A–D** γδ^+^ T cells were distributed in the periphery of the infiltrate, irrespective on whether the patients had moderate (5 patients) or high (2 patients) amounts of tumor-infiltrating γδ T lymphocytes.

In the peripheral blood of healthy adult individuals, the majority of γδ T cells expresses the Vδ2 TCR, whereas cells carrying Vδ1 dominate among intraepithelial γδ T cells [23].

Vδ1^+^ T cells were the predominant melanoma-infiltrating γδ T cell population in 23 patients (50%), while cells expressing Vδ2 predominated in 19 patients (42%), and 4 patients (8%) had equal percentages of Vδ1^+^ and Vδ2^+^ cells (**Figure 2E**). This was not due to a bias in the overall γδ T cell population in melanoma patients, as the Vδ1 to Vδ2 ratio in their peripheral blood was low and similar to that found in healthy individuals (**Figure 2E**).

**Figure 3:**
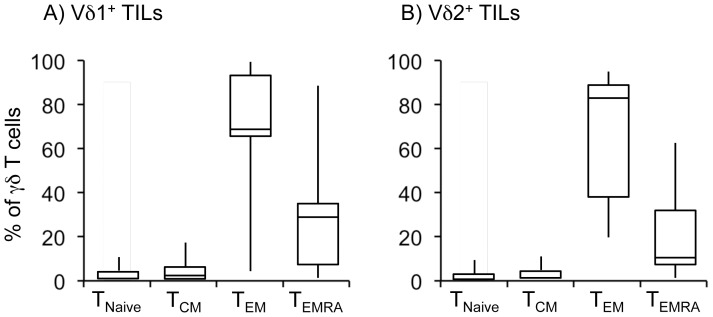
Memory and effector phenotypes of Vδ1 and Vδ2 T cells in TILs of patients with melanoma. TILs from patients with melanoma were obtained as described under Materials and Methods and were stained with anti-Vδ1, anti-Vδ2, anti-CD45RA and CD27 mAbs. Percentages of T_naive_ (CD45RA^+^CD27^+^), T_CM_ (CD45RA^−^CD27^+^), T_EM_ (CD45RA^−^CD27^−^) and T_EMRA_ (CD45RA^+^CD27^−^) cells were determined by FACS analysis. Shown are cumulative data for Vδ1 and Vδ2 T cells.

Human γδ T cells include those with naive or central-memory phenotypes (T_naive_, CD45RA^+^CD27^+^; T_CM_, CD45RA^−^CD27^+^) that home to secondary lymphoid organs, but that lack immediate effector function, and those with effector-memory (T_EM_, CD45RA^−^CD27^−^) and terminally-differentiated (T_EMRA_, CD45RA^+^CD27^−^) phenotypes that home to sites of inflammation and that display immediate effector functions as cytokine production and cytotoxic activity [40]. We studied the memory subset distribution of the infiltrating γδ T cells, assessing the expression of CD27 and CD45RA on Vδ1^+^ and Vδ2^+^ cells. The analysis showed, that the majority of melanoma-infiltrating Vδ1^+^ T cells had a T_EM_ (64.9%) and T_EMRA_ (26.1%) phenotype, whereas T_naive_ and T_CM_ accounted for 2.3% and 5.3%, respectively (**Figure 3A**). Similarly, the melanoma infiltrating Vδ2 T cells were predominantly T_EM_ (84.5%) and T_EMRA_ (10.4%). while T_naive_ and T_CM_ subpopulations accounted for 1.2% and 3.7%, respectively (**Figure 3B**).

**Figure 4:**
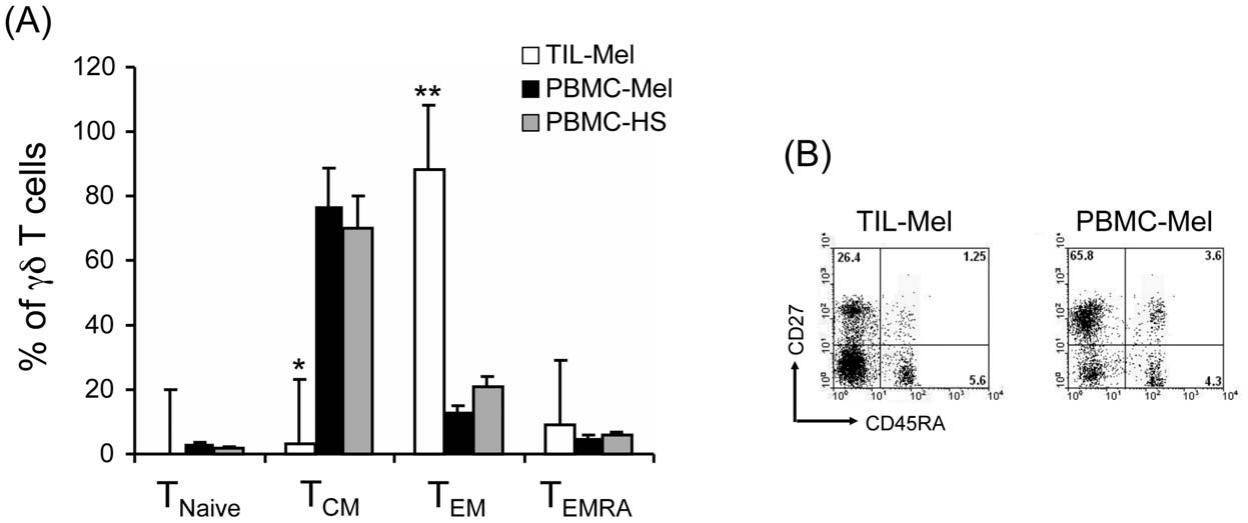
Comparison of Vδ2 T cell subset distribution in TILS and PBMCs form patients with melanoma and healthy individuals. TILs from patients with melanoma (TIL-Mel) and PBMCs from patients with melanoma (PBMC-Mel) and healthy subjects (PBMC-HS) were obtained as described under Materials and Methods and were stained with anti-Vδ2, anti-CD45RA and CD27 mAbs. Percentages of T_naive_ (CD45RA^+^CD27^+^), T_CM_ (CD45RA^−^CD27^+^), T_EM_ (CD45RA^−^CD27^−^) and T_EMRA_ (CD45RA^+^CD27^−^) cells were determined by FACS analysis. (A) Shows cumulative data for Vδ2 T cells and (B) shows representative flow cytometry panels from a healthy subject and a patient with melanoma, upon gating on Vδ2^+^ cells and staining with CD27 and CD45RA. *p<0.001 and **p<0.01 when compared to values in PBMCs.

The finding that the majority of melanoma-infiltrating Vδ1^+^ T cells had a T_EM_ and T_EMRA_ phenotype is not surprising as this is the phenotype of circulating Vδ1^+^ T cells. However, the detection of Vδ2^+^ T cells with a T_EM_ and T_EMRA_ phenotype is intriguing as circulating Vδ2^+^ T cells are predominantly T_CM_ and T_EM_ [41]. To understand if the predominance of tumor-infiltrating Vδ2^+^ T cells with a T_EM_ and T_EMRA_ phenotype was due to the tumor microenvironment or simply reflected an overall bias in melanoma patients, we studied the phenotype of γδ T cells in the peripheral blood of 15 healthy subjects and 46 melanoma patients, and compared them with the infiltrating γδ T cells. As shown in **Figure 4A**, Vδ2^+^ T cells obtained from peripheral blood of healthy subjects showed a predominant T_CM_ phenotype (76.8%), and similarly did circulating Vδ2^+^ γδ T cells from melanoma patients (69.6%). Conversely, the T_CM_ cell subset was very poorly represented in the infiltrating γδ T cell population (approximately 3.7%).

An opposite pattern was found for Vδ2^+^ γδ T cells with a T_EM_ phenotype, which were poorly represented amongst circulating Vδ2^+^ γδ T cells of healthy subjects (13%) and melanoma patients (21%), while being the predominant population (84.5%) in the melanoma-infiltrating γδ T cells.

These results clearly demonstrate that the cells committed to effector activities at the tumor site are phenotypically different respect to circulating Vδ2^+^ γδ T cells. **Figure 4B** shows a typical FACS analysis of one representative sample, indicating the different phenotype of Vδ2^+^ γδ T cells in each analyzed group.

### Cytokine Production and Cytotoxic Activity of Melanoma-infiltrating γδ T Cells

**Figure 5:**
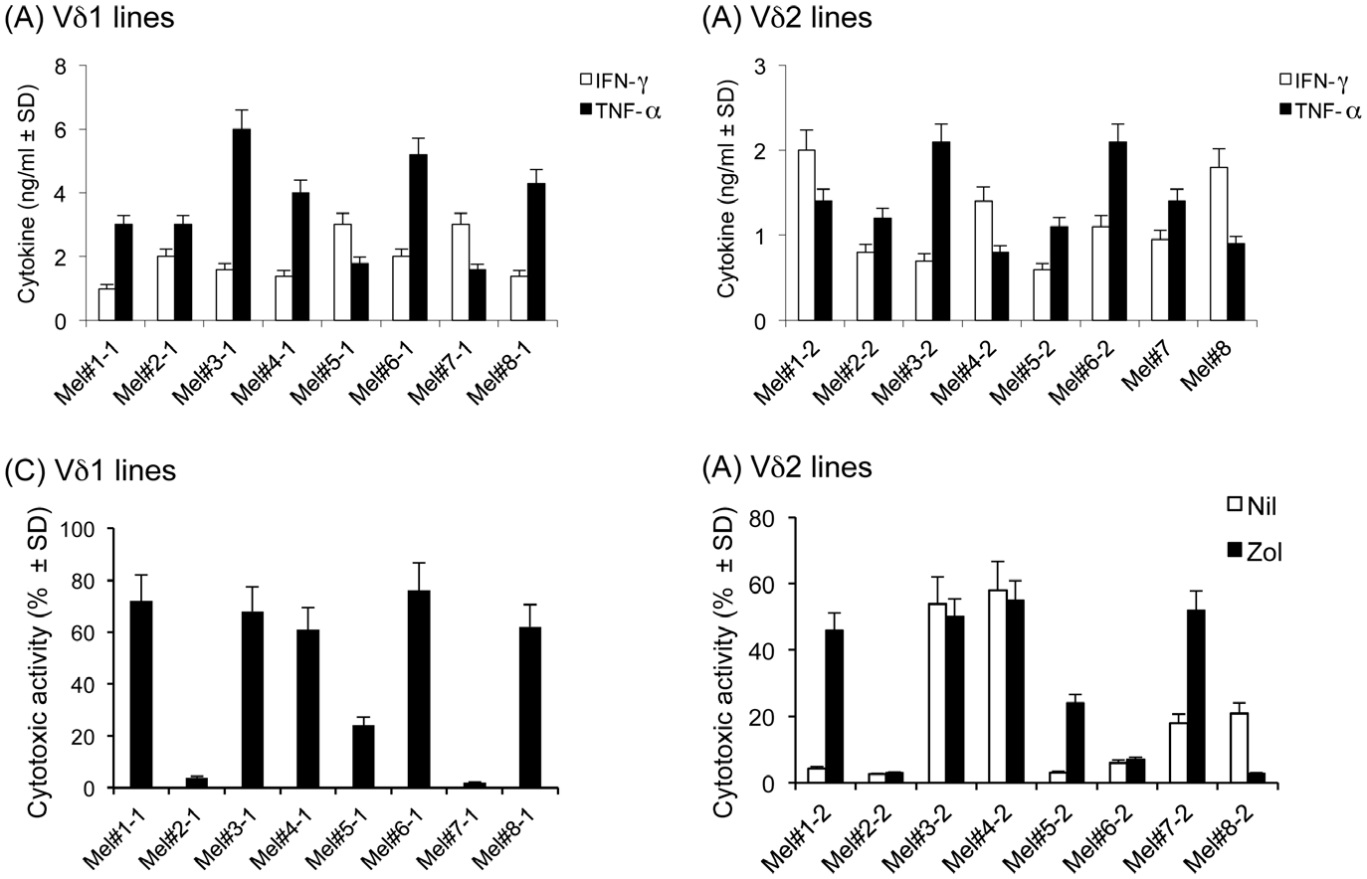
Cytokine production capacity and cytotoxic activity of melanoma-derived γδ T cell lines. Cytokine production by melanoma-derived Vδ1 (A) and Vδ2 (B) cell lines was assessed by ELISA analysis of supernatants after 24-hrs stimulation with PMA and ionomycin. Cytokines were <1 ng/ml in the supernatants of unstimulated Vδ1 and Vδ2 T cell cultures. Values are the mean from duplicate wells. The cytotoxic activity of melanoma derived Vδ1 (C) and Vδ2 (D) T cell lines was analyzed for against the autologous melanoma cell line A375 at different E:T cell ratios. In experiments shown in (D), A375 target cells were pre-treated with zoledronate, as described under Materials and Methods. Shown are data from a representative experiment with 8 different T cell lines, at an E:T ratio of 25∶1.

The yield of primary γδ T cells isolated from melanomas did not allow a detailed investigation; therefore, functional studies were performed with a panel of Vδ1^+^ and Vδ2^+^ polyclonal γδ T cell lines (see also Materials and Methods) derived from tumor-infiltrating immune cells of 8 melanoma patients. All tested Vδ1^+^ and Vδ2^+^ T cell lines produced very high levels of the proinflammatory cytokines TNF-α and IFN-γ after stimulation with PMA and ionomycin (**Figure 5A and B**). To address the potential role of cutaneous γδ T cells in tumor surveillance, their ability to mount cytolytic responses against melanoma cells was examined. Tumor-derived, polyclonal Vδ1^+^ and Vδ2^+^ T cell lines were tested in cytotoxicity assays using heterologous melanoma cell lines as targets. Of the eight tested Vδ1^+^ T cell lines, five exerted robust cytotoxic responses against the melanoma cell line A375, resulting in up to 70% target cell death at an E:T cell ratio of 25∶1; one line had poor, yet detectable cytotoxic activity, whereas two lines failed to kill (**Figure 5C**).

The cytotoxic capacity of melanoma-derived, polyclonal Vδ2^+^ T cell lines was examined in similar experimental conditions. Remarkably, and in clear contrast to Vδ1^+^ T cell lines, only two out of eight tested Vδ2^+^ T cell lines demonstrated clear cytotoxic activity against A375 melanoma cells, with an average of 75% target cell lysis at an E:T cell ratio of 25∶1 (**Figure 5D**); two cell lines had low cytotoxic activity, while four lines completely failed to kill. However, pre-treatment of the A375 melanoma target cells with zoledronate, a drug shown to sensitize several tumor targets to the cytolytic activity of Vδ2^+^ T cells, was able to increase their killing by Vδ2^+^ T cell lines in four instances (**Figure 5C**).

### Clinical Correlates and Statistical Considerations

γδ T cells were found in 46 of the 74 patients (62.0%). The success rate of γδ T cells isolation ranged from over 60% in patients at stage 0 or I, to 86% in patients at stage II, 42% in patients at stage III and 0% for patients in stage IV. When data were cumulated for patients with localized melanoma or disseminated disease (e.g., absence or presence of metastases), γδ T cells were recovered in 71.9% of non-metastastic patients (stage 0-I-II), and in 29.4% of patients with metastases (stage III and IV) (**Table 1**). We analyzed the statistical significance of the association between γδ cells isolation rate in tumor-infiltrating immune cells and the stage of disease. The standard non-parametric approach exploited when outcomes are binary relies on the Kendall’s Tau-b. In our sample this association is equal to −0.367 with an asymptotic standard error (ASE) of 0.112, indicating that γδ T cells and the cancer’s stage are negatively correlated. However to check whether this association is statistically significant or rather it is simply due to sampling variations, we tested the null hypothesis of no association using the Pearson χ^2^ test. This is asymptotically distributed as χ^2^ distribution with 1 df. In our study, the Pearson χ^2^ is equal to 10.06 with a p-value of 0.002. Thus the null hypothesis is rejected at 1% statistical significant level. For robustness of statistical significance, we have also computed the LR χ^2^ statistic and the Fischer’s exact test. Generally these tests perform better than the Pearson χ^2^ test when cell frequencies are small. Also in this case the LR χ^2^ test (p-value of 0.002) and the Fisher’s exact test (p-value 0.003) rejects the null of no association even at 1% significance level. Finally we have also run a simple logit model to estimate the odds-ratio between cancer’s stage and γδ T cell isolation rate. The odds ratio is 0.16 (SE = 0.10).

**Table 1:** Association between rate of γδ T cells and clinical features in patients with melanoma.

Feature	γδ T cells isolation rate	γδ T cells (% ± SE)	Vδ1 T cells (% ± SE)	Vδ2 T cells (% ± SE)
**Stage (TNM system)**				
**0**	6/9 (66.7%)	1.72±1.07	1.21±0.73	0.60±0.41
**I**	16/26 (62.5%)	1.52±0.81	0.94±0.61	0.62±0.42
**II**	19/22 (86.3%)	1.69±1.03	0.97±0.64	0.71±0.41
**III**	5/12 (41.7%)	0.97±0.82	0.89±0.71	0.22±0.23*
**IV**	0/5 (0%)	0±0	0±0	0±0
**0-I-II**	41/57 (71.9%)	1.62±0.93	0.98±0.65	0.67±0.42
**III-IV**	5/17 (29.4%)	0.67±0.72	0.63±0.61	0.17±0.17**
**Total**	46/74 (62.1%)			

The rate of γδ T cell isolation was calculated by dividing the number of patients from with γδ T cells were isolated by the total number of patients.

* p = 0.038 when compared to values at stage 0, p = 0.026 when compared to values at stage I and p = 0.013 when compared to values at stage II.

** p = 0.00026 when compared to values at stages 0-I-II.

The *prima facie* inverse correlation between melanoma stage and the recovery rate of γδ T cells was provocative, and was investigated further. As shown in **Table 1**, percentages of total γδ T cells and their Vδ1 subset, among infiltrating lymphomonocytes, were lower in patients at stages III and IV, even if values at stage III were not significantly different from values at stages 0, I and II. Also percentages of Vδ2 cells were lower in patients at stage III and IV, but values at stage III attained statistical significance as compared to patients at stage 0, I and II. When data were cumulated for patients with localized melanoma or disseminated disease (e.g., absence or presence of metastases), there was a clear trend whereby percentages of total γδ T cells and their Vδ1 and Vδ2 subsets declined in patients with advanced disease, but again only differences in percentages of Vδ2 T cells were significantly different. These results indicate that percentages of tumor-infiltrating Vδ2 T cells are inversely correlated with the stage of disease in patients with melanoma.

## Discussion

Substantial evidence indicates that the immune system participates in cancer pathogenesis and may contribute to either disease progression or inhibition of tumor growth. It is common for regressing tumor lesions to be markedly infiltrated by mononuclear cells and the presence of T cells has been associated with improved prognosis in patients with different types of carcinomas [8]–[10]. Relevant to this study, patients with lymphocytic infiltrate in primary melanomas had lower risk of metastasis and death than patients with sparse or absent infiltrate [4], [5].

γδ T lymphocytes are important effector cells of the immune system that play a role in the anti-tumor surveillance in the periphery. For instance, murine skin harbors a major population of γδ T cells, known as dendritic epidermal T cells (DETCs), which appear to play a critical role in tumor surveillance, as evidenced in γδ T cell-deficient mice [22]. However, a counterpart of DETCs does not exist in human epidermis, and it is thus unclear what types of lymphocyte, if any, mediate equivalent immune surveillance functions in human skin.

Results herewith reported show that γδ T cells are the major lymphocyte population infiltrating melanoma, and both Vδ1^+^ and Vδ2^+^ cells are involved. This finding is not surprising as it recapitulates the microenvironmental conditions occurring under physiologic conditions *in vivo* in humans. In fact, Vδ1^+^ T cells are the prevalent γδ T cell population in human tissues including intestinal epithelium and skin [42]. However, our study also demonstrate the presence of Vδ2^+^ T cells among melanoma-infiltrating immune cells, which are the major γδ T cell population in approximately 40% of patients. Given that Vδ2^+^ T cells are absent from normal skin [42], their presence in the context of melanoma is highly suggestive of a migratory process from the blood or lymphoid tissues. Accordingly, more than 90% of melanoma-infiltrating Vδ2^+^ T cells have a T_EM_ and T_EMRA_ phenotype, indicative of cells that home to sites of inflammation and that display immediate effector functions as cytokine production or cytotoxic activity [41]. Very recently, a distinct subset of proinflammatory CLA^+^, CCR6^+^ Vγ9Vδ2 T cells has been described which is rapidly recruited from the blood to the skin compartment [43], but we do not have evidence if the Vγ9Vδ2 T cells which infiltrate melanoma belong to this novel γδ T cell subset.

Conversely, circulating Vδ2^+^ T cells from the same patient have a predominant T_CM_ phenotype, similarly to that occurring in healthy subjects, and that identifies cells that home to secondary lymphoid organs, but that lack immediate effector function.

Collectively, these results strongly indicate that while Vδ1^+^ T cells may be activated locally in the tumor microenvironment, Vδ2^+^ T cells are most likely recruited from the peripheral blood or secondary lymphoid organs by virtue of a migratory process orchestrated by chemokines or other inflammatory molecules produced at the tumor site. Both the resident Vδ1^+^ and the migrated Vδ2^+^ T cells may contribute to control tumor growth, as suggested by their ability to produce cytokines with proven anti-tumor activity (TNF-α and IFN-γ) and to kill melanoma cell targets *in vitro*. However, substantial differences were found in the cytotoxic capability of Vδ1^+^ and Vδ2^+^ T cells: while the majority of Vδ1^+^ T cell lines exerted cytotoxic activity against the melanoma cell line A375, only two out of eight tested Vδ2^+^ T cell lines were able to kill this tumor target cell. Moreover, treatment with zoledronate was able to sensitize melanoma cell targets to Vγ9Vδ2 T cell cytotoxicity.

It has been demonstrated that treatment of tumor cells with zoledronate leads to the intracellular accumulation of phosphoantigens (typically IPP), thus favoring recognition and killing of tumor cells by the reactive Vγ9Vδ2 T lymphocytes [44], [45]. How far other molecules, such as the ectopically expressed F1-ATPase, which has been claimed to serve as the Vγ9Vδ2 TCR ligand expressed by tumor cells [46], are involved in IPP recognition remains unclear.

In our study, the presence of γδ cells in tumor-infiltrating immune cells correlates with early stage melanomas as indicated by higher rate of γδ T cells isolation and higher percentages of γδ T cells in patients at early stage of development of melanoma (stage 0-I-II) and without metastasis. Of note, γδ cells were scarcely found or absent in patients at an advanced stage (stage III and IV) and in metastatic melanomas (71.9% non metastatic vs 29.4% metastatic) and this was particularly evident for Vδ2 T cells which showed an inverse correlation with the stage of melanoma, a finding which could be associated with a poor effectiveness of the local immune response at advanced phases of tumor progression. Recent studies have shown a high frequency of γδ T cells among TILs from patients with different types of cancer, but the clinical relevance of these intratumoral γδ T cells is unknown. In contrast to the present study, percentages of γδ T cells within renal cell carcinoma fail to correlate with any prognostic features [47] and intratumoral γδ T cell numbers are positively correlated with advanced tumor stages, HER2 expression status, and high lymph node metastasis but inversely correlated with relapse-free survival and overall survival of breast cancer patients [48]. Actually we can only speculate on the discrepancy between melanoma and renal and breast cancer. γδ cells comprise two major subsets (Vδ1 and Vδ2) with different memory/effector phenotypes and heterogeneous functional properties that can be discerned by the use of additional markers (cytokine production, cytotoxic activity, migratory properties); hence positive or negative correlation with prognosis may depend on the specific γδ subset recruited at the tumor site. Furthermore, the net biologic effects of γδ T cells may depend on the tumor type and the tumor site, perhaps reflecting microenvironmental differences.

The analysis of TIL responses to cancer antigens has served to define many of the relevant TAs, and adoptive cellular immunotherapy with TILs has achieved some of the most remarkable immunotherapy results to date [13], [49]. For instance, high clinical response rates have been obtained in metastatic melanoma patients with TIL-based protocols in combination with non-myeloablative lymphodepleting chemotherapy (cyclophosphamide and fludarabine) immediately prior or added to infusion of TIL and high-dose IL-2 therapy [11], [13].

Our study, showing that γδ T cells are found in the majority of melanoma-infiltrating lymphocyte populations, and they recognize and kill melanoma cells, suggest that a natural immune response mediated by these lymphocytes may contribute to the immunosurveillance of melanoma. Such observations may foster the development of novel alternative or adjuvant therapies targeting γδ T cells for the treatment of melanoma patients. This might be achieved for instance by stimulating γδ T cells in these patients through injection of zoledronate and IL-2, as recently performed by our group in prostate and breast cancer [50], [51]. Alternatively, it could be achieved by adoptive transfer of *ex vivo* expanded autologous γδ T cells derived from cancer patients [52], [53]. Both the γδ T cell transfer and the infusion of bisphosphonates have been proven to be well tolerated [50], [53].

In conclusion, it can be speculated that the *in vitro* expansion of γδ T cells and the subsequent infusion of these cells plus zoledronate, in combination with other antitumor agents may be of significant clinical benefit in the treatment of melanoma; in turn, this could allow us to extend the life span of patients and thereby to increase the window of the patients’ availability for other more specific molecular approaches.
